# Expert consensus on treating HR+/HER2- metastatic breast cancer based on real-world practice patterns observed in the RETRACT survey of US oncologists

**DOI:** 10.1016/j.breast.2025.104485

**Published:** 2025-05-03

**Authors:** Hope S. Rugo, Aditya Bardia, William J. Gradishar, Erika P. Hamilton, Sara A. Hurvitz, Komal Jhaveri, Reshma Mahtani, Sara M. Tolaney

**Affiliations:** a University of California San Francisco Helen Diller Family Comprehensive Cancer Center, San Francisco, CA, USA; b UCLA Health Jonsson Comprehensive Cancer Center, Los Angeles, CA, USA; c Northwestern University Feinberg School of Medicine, Chicago, IL, USA; d Sarah Cannon Research Institute, Nashville, TN, USA; e University of Washington Fred Hutchinson Cancer Center, Seattle, WA, USA; f Memorial Sloan Kettering Cancer Center, New York, NY, USA; g Miami Cancer Institute, Baptist Health South Florida, Miami, FL, USA; h Dana-Farber Cancer Institute, Boston, MA, USA

**Keywords:** Metastatic breast cancer, Hormone receptor, Human epidermal growth factor receptor 2, Treatment patterns, Practice guidelines, Side effects, Biomarker testing

## Abstract

Hormone receptor-positive, HER2-negative metastatic breast cancer (HR+/HER2-mBC) is incurable, but recent progress has been made in developing new treatment options and the treatment landscape is rapidly shifting. There are published recommendations for treatment choices and sequencing to help guide oncologists in treating HR+/HER2-mBC, but little evidence has been published regarding real-world practice patterns. The REal-world TReatment patterns And Considerations of Toxicity in HR+/HER2-mBC (RETRACT) survey was designed to evaluate real-world practice patterns in the testing and management of this disease by US oncologists. The survey questions were answered via an online platform and the data were anonymized before analysis. A total of 150 oncologists practicing at academic and community centers completed the survey. The results showed this sample of oncologists largely followed recommended best practices for testing biomarkers, selecting treatments, and managing adverse events. However, several items did show substantial minorities of oncologists not in alignment with recommendations in areas including the definition and treatment of visceral crisis, ideal treatment for patients with endocrine resistance, the routine use of next-generation sequencing for biomarker testing, and the use of prophylactic measures for treatment-related adverse events in patients receiving alpelisib.

## Highlights

• Reported treatment patterns are largely aligned with published guidelines.
• Some respondents did not use of next-generation sequencing to assess biomarkers.
• Use of prophylactic measures for treatment-related adverse events was not universal.

## 1 Introduction

Breast cancers (BC) are classified based on distinct molecular characteristics, including the expression of estrogen or progesterone receptor (HR) and whether it overexpresses human epidermal growth factor 2 (HER2). Tumors that are HR-positive and negative for HER2 overexpression (HR+/HER2-) account for about 70% of all cases of BC [1]. Metastatic breast cancer (mBC) remains incurable, but progress has been made in developing new treatment options [2]. The treatment landscape for HR+/HER2-mBC is therefore rapidly shifting.

Guidelines recommend treatment with multiple lines of endocrine therapy (ET) with or without targeted treatments until all options have been exhausted [3,4]. ET combined with targeted therapies are recommended for first-line therapy for HR+/HER2-mBC including aromatase inhibitors (AI) or fulvestrant combined, a selective estrogen receptor degrader (SERD), with cyclin-dependent kinase 4/6 inhibitors (CDK4/6i) such as ribociclib, abemaciclib, or palbociclib [3,4]. Phase 3 trials of CDK4/6is showed all were associated with comparable and significant improvements in progression-free survival (PFS) when used in the first- or second-line setting [[5], [6], [7], [8], [9], [10], [11], [12], [13], [14], [15]]. However, only ribociclib has demonstrated significant improvement in patient overall survival (OS) [13], while clinically meaningful, but not statistically significant improvement in OS was reported with abemaciclib [16].

Targeted second-line therapies for HR+/HER2-mBC include alpelisib in patients with activating mutations in tumor *PIK3CA*, which significantly improved PFS in patients with *PIK3CA* mutations who received alpelisib plus fulvestrant compared to placebo plus fulvestrant [17,18]. Capivasertib was approved as a second-line treatment in November 2023 for use in patients with HR+/HER2-locally advanced or metastatic BC with one or more *PIK3CA*/*AKT*/*PTEN* alterations [19] based on results from the phase 3 CAPItello-291 trial (NCT04305496) [20]. An oral SERD, elacestrant, received Food and Drug Administration (FDA) approval for second-line use in HR+/HER2-locally advanced or metastatic BC with *ESR1* mutations in January 2023 [21] based on data from the phase 3 EMERALD trial (NCT03778931) [22].

For patients without any targetable mutations, the combination of everolimus plus exemestane as second-line treatment is recommended by published guidelines [3,4] and significantly improves PFS but not OS [[23], [24], [25]]. Everolimus plus fulvestrant also significantly improves PFS in first- and second-line settings [26,27].

Chemotherapy antibody-drug conjugates (ADCs) can also be considered following progression on ET. ADCs have demonstrated improvements in PFS and/or OS in mBC in pretreated patients including trastuzumab deruxtecan (T-DXd) [28,29], sacituzumab govitecan [30,31], and datopotamab deruxtecan (Dato-DXd) [32].

In addition to navigating multiple lines of treatments, oncologists must successfully manage and mitigate treatment-associated adverse events (AEs) that frequently arise during the care of patients with mBC. Treatment related side effects are common with the use of ET, targeted therapies, ADCs, as well as chemotherapies [17,20,[33], [34], [35], [36]], and these require nuanced and proactive management to optimize treatment outcomes.

Clearly, many treatment options based on published guidelines are available for HR+/HER2-mBC. However, data regarding real-world practice patterns are lacking, and substantial variability is to be expected as clinicians navigate the guidelines considering individual patients with their varying context of comorbidities, treatment history, the presence of targetable mutations, and more. The REal-world TReatment patterns And Considerations of Toxicity in HR+/HER2-mBC (RETRACT) survey was developed to evaluate real-world practice patterns in the testing and management of HR+/HER2-mBC by US oncologists.

## 2 Materials and methods

A Steering Committee of clinicians who are experts in the treatment of breast cancer was recruited through recommendations by the Chair to design the RETRACT survey to obtain detailed information about the real-world practice patterns of oncologists (Supplemental Fig. S1). It was distributed among practicing oncologists in academic centers and community centers across the US. Eligible oncologists who agreed to complete the survey were compensated for their time. The questionnaire was accessed via an online platform (eScientiq, Cambridge, MA) and included questions about diagnosis and treatment of their patients with mBC along with management of common toxicities associated with current treatments. Subsequently, the Steering Committee used the eScientiq platform to form a consensus on the following interpretations and recommendations. Ethical review was not required because the survey was anonymized.

The eligibility of survey respondents was restricted to those who self-reported as having experience treating patients with HR+/HER2-mBC. Respondents were also required to be US clinical oncologists practicing in community or academic settings. The survey was disseminated to oncologists from academic and community medical centers using internal and commercially available databases. Responses were accepted from December 1, 2023, through April 30, 2024. Surveys were accepted as completed if ≥ 50% of the items were answered, although a majority answered all.

The survey results were anonymized before processing. All responses were analyzed using descriptive statistics and reported as absolute frequencies, percentages, and means, as appropriate.

## 3 Results

### 3.1 Responder demographics

The survey was sent to approximately 1000 oncologists who were working at academic or community medical centers in the US. A total of 187 respondents registered on the digital platform, and 150 completed the survey. Characteristics of the survey respondents and their patients are summarized in Table 1. About one-third (50/146; 34%) worked primarily in a community setting, primarily cancer clinics and outpatient cancer care centers. The number of patients with breast cancer seen per month ranged from 25 or fewer (9/146; 6%) to over 200 (23; 16%). Over 8 in 10 respondents (118/146; 81%) indicated the proportion of their patients with HR+/HER- BC who have metastatic disease is 40% or less.

**Table 1:** Characteristics of respondents and their patients.

Work setting (n = 146)	n (%)

• Academic	96 (66)
• Community Center	50 (34)
Number of patients with breast cancer seen monthly (n = 146)	n (%)

0–25	9 (6)
26–50	25 (17)
51–100	32 (22)
101–150	42 (29)
151–200	15 (10)
>200	23 (16)
Proportion of patients with breast cancer who have HR+/HER2- (n = 143)	n (%)

0–20%	3 (2)
21–40%	6 (4)
41–60%	40 (28)
61–80%	88 (62)
>80%	6 (4)
Proportion of patients with HR+/HER2- breast cancer who have metastatic disease (n = 146)	n (%)

0–20%	50 (34)
21–40%	68 (47)
41–60%	25 (17)
61–80%	2 (1)
>80%	1 (1)

### 3.2 Biomarker testing

**Fig. 1:**
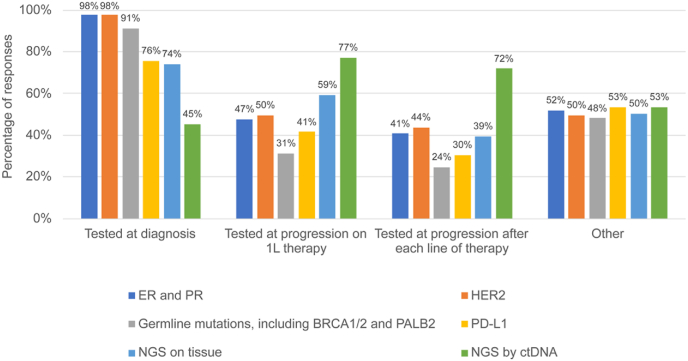
Survey question – “Please indicate which biomarkers you test for in patients with mBC and the times at which you test for these biomarkers.” Multiple answers were permitted (n = 135).

The survey results regarding biomarker testing are summarized in Fig. 1. It shows that virtually all respondents (98%) reported testing for ER/PR and HER2 status at diagnosis, while substantial majorities also tested at diagnosis for germline mutations (91%), including *BRCA1/2* and *PALB2*, tumor PD-L1 expression (76%), and next generation sequencing (NGS) on tumor tissue (74%). A greater number of respondents used NGS with circulating tumor DNA (ctDNA) after disease progression on treatment (first-line: 67%; second-line: 72%). Asked what proportion of patients with mBC they test with NGS, 103/142 respondents (73%) tested more than 80% of their patients with mBC, while 39/142 (27%) used NGS testing in 80% or less of patients with mBC.

### 3.3 First-line therapy

The first-line therapies that survey respondents prescribed for patients with HR+/HER2-mBC are summarized in Table 2. As initial therapy, 113/143 (79%) physicians reported utilizing CDK4/6i plus an AI and reported using this combination in over 60% of newly diagnosed patients. The first-line CDK4/6i was ribociclib in 93/139 (67%) of responses.

**Table 2:** What percentage of patients in your practice with HR+/HER2-mBC receive the following first-line treatments? (n = 143).

Treatment	Percentage receiving in first line	Number of respondents that chose this option (%)
CDK4/6i + AI	0%–40%	9 (6.3)
	41%–60%	21 (14.7)
	61%–100%	113 (79.0)
	No entry	0 (N/A)
CDK4/6i + fulvestrant	0%–40%	119 (83.2)
	41%–60%	14 (9.8)
	61%–100%	4 (2.8)
	No entry	0 (N/A)
ET monotherapy	0%–40%	127 (88.8)
	41%–60%	3 (2.1)
	61%–100%	0 (0)
	No entry	2 (1.4)
Conventional Chemotherapy	0%–40%	127 (88.8)
	41%–60%	0 (0)
	61%–100%	2 (1.4)
	No entry	1 (0.7)
Other (e.g., radiation, ADCs, etc.)	0%–40%	119 (83.2)
	41%–60%	0 (0)
	61%–100%	4 (2.8)
	No entry	2 (1.4)

#### 3.3.1 CDK4/6 inhibitors after progression on a CDK4/6 inhibitor plus ET

**Fig. 2:**
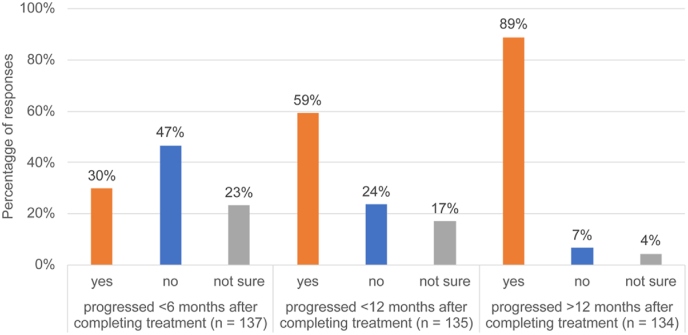
Survey question – “If a patient with HR+/HER2-mBC received a CDK4/6i in the adjuvant setting and progressed after completing treatment (while still on adjuvant ET), would you prescribe CDK4/6i + a different ET in the 1L mBC setting?” Respondents answered this question for progression at 3 different timepoints after completing treatment: <6 months (n = 137), <12 months (n = 135), and >12 months (n = 134).

A majority of respondents (90/137; 66%) reported they would not prescribe first-line CDK4/6i plus ET to patients who had a disease-free interval of less than 6–12 months after an adjuvant CDK4/6i-containing therapy. In a separate survey question, respondents were queried as to whether they would treat a patient with CDK4/6i plus a different ET if patients had PD < 6 months, <12 months, or >12 months after completing treatment with CDK4/6i in the adjuvant setting (Fig. 2). Respondents were more likely to prescribe CDK4/6i plus ET in patients with a longer progression-free interval after completing adjuvant CDK4/6i treatment, including 119/134 (89%) if progression occurred more than 12 months later. In addition, most would use a different CDK4/6i in patients who had PD less than 6 months after previous CDK4/6i treatment (121/129; 94%) or after less than 12 months (121/132; 92%). However, more respondents were willing to retry the same CDK4/6i if a patient had PD more than 12 months after completing the previous CDK4/6i treatment (31/134; 23%), although the majority still preferred to use a different CDK4/6i (92/134; 69%).

#### 3.3.2 First-line therapy in patients with visceral crisis

The RETRACT survey asked participants to select the option that best represented their understanding of visceral crisis from a list of seven possibilities, and 89/145 (61%) chose the definition aligned with the ABC5 definition, “Severe organ dysfunction, as assessed by signs and symptoms, laboratory studies, and rapid progression of disease.” [37] In addition, 27/145 (19%) chose “liver metastases with liver dysfunction,” which would also qualify under the ABC5 definition. In addition, 59/137 respondents (43%) reported they would not prescribe ET plus CDK4/6i to patients in visceral crisis.

### 3.4 Second-line therapy

**Fig. 3:**
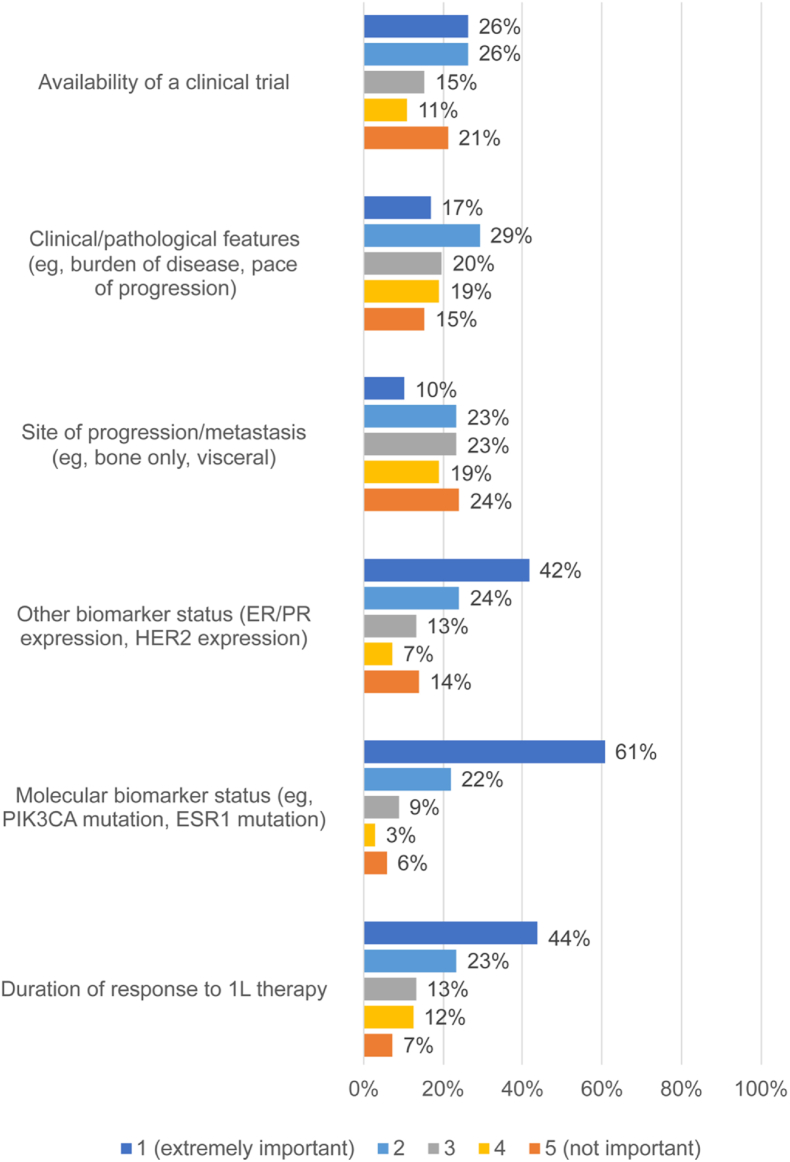
Respondents rated the importance of several factors for determining the best second-line treatment option for patients with mBC on a scale of 1–5 (1 = extremely important; 5 = not important). (n = 137).

Survey respondents were asked to rate the importance of 6 different factors in determining the choice of second-line therapy for patients with HR+/HER2-mBC (Fig. 3), and the 2 factors most frequently rated as extremely important were: molecular biomarker status (83/137; 61%), and duration of response to first-line therapy (60/137, 44%). In addition, 124/135 (92%) usually tried to exhaust all possible ET-based treatment options (with or without targeted agents) before transitioning patients to chemotherapy or ADCs.

**Fig. 4:**
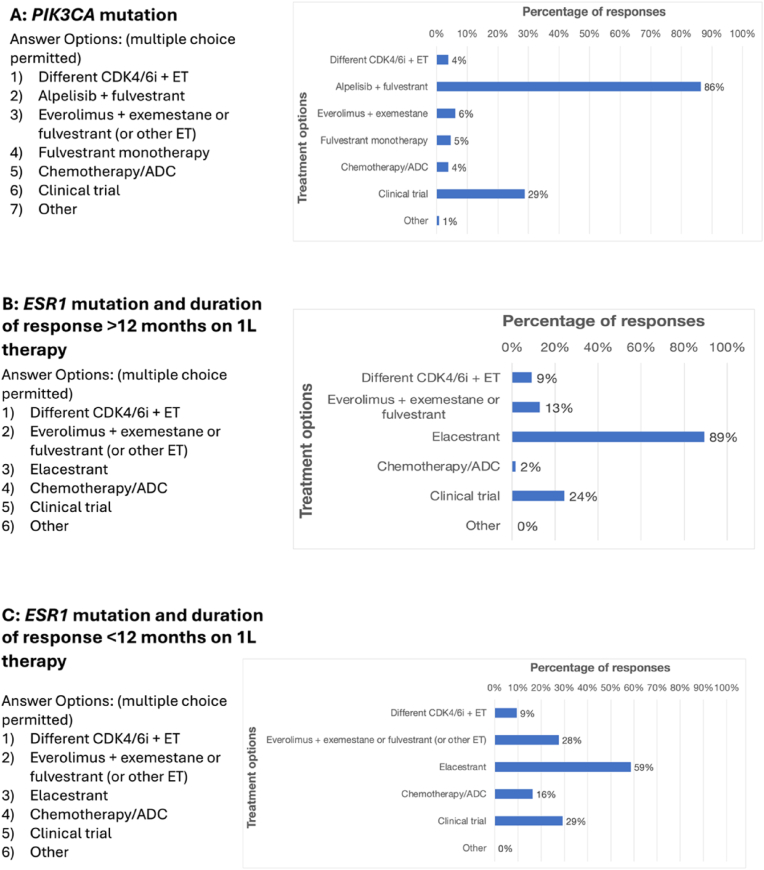

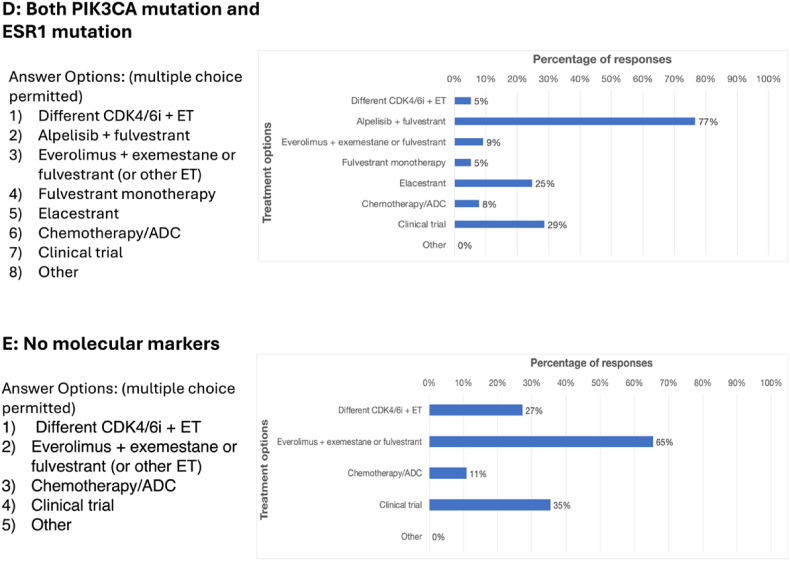
Respondents indicated which treatments they preferred to use in the second-line setting for several different mBC tumor types, assuming the patient received first-line CDK4/6i + ET. Multiple answers were permitted. **A)***PIK3CA* mutations (n = 132). **B)***ESR1* mutations and a duration of response of >12 months on first-line therapy (n = 132). **C)***ESR1* mutations and a duration of response of <12 months on first-line therapy (n = 116). **D)** Both *PIK3CA* and *ESR1* mutations (n = 77). **E)** No actionable molecular biomarkers (n = 110).

RETRACT included several questions regarding 5 different cases of HR+/HER2-mBC that differed in their mutational status and responses to first-line therapy (Fig. 4). Survey respondents were asked to choose their preferred second-line treatment for each case, assuming the patient received a CDK4/6i plus ET in the first line. For patients with mutated *PIK3CA*, the most frequently chosen second-line treatment was alpelisib plus fulvestrant (114/132; 86%). The survey did not include capivasertib plus fulvestrant as an option since it was not yet approved when RETRACT was developed, but 40/138 (29%) entered this regimen in the free text field, unprompted.

Most respondents (118/132; 89%) chose elacestrant for patients with mutated *ESR1* who had a duration of response (DOR) of more than 12 months to first-line CDK4/6i plus ET. However, for a patient with mutated *ESR1* and a DOR in the first line of less than 12 months, the responses were more split, with 68/116 (59%) selecting elacestrant and 32/116 (28%) choosing everolimus plus exemestane, fulvestrant or other ET. For patients with both *PIK3CA* and *ESR1* mutations, a majority chose alpelisib plus fulvestrant (60/77; 78%), and 17/77 (22%) expressed a preference for capivasertib plus fulvestrant in the free text field.

Finally, in patients with no molecular biomarkers present at the time of second-line therapy, most respondents chose everolimus plus exemestane, fulvestrant, or other ET (72/110; 65%).

### 3.5 Managing treatment-associated adverse events

#### 3.5.1 Endocrine therapy

Nearly all survey respondents reported switching patients to a different ET as a strategy they use to manage intolerable AEs associated with ET (127/134; 95%), which may include, but are not limited to, hot flashes, sexual dysfunction, weight gain, musculoskeletal symptoms, and fatigue. Over three-fourths of respondents (105/134; 78%) used non-steroidal anti-inflammatory drugs and over-the-counter medications for ET-associated AEs, and 90/134 (67%) used the anti-depressant duloxetine. In free text responses, 25/134 respondents (19%) indicated using acupuncture in such cases, and 15/134 (11%) listed exercise.

#### 3.5.2 Nausea

Regarding their management of treatment-associated nausea, most respondents reported using olanzapine (126/134; 94%), corticosteroids (102/134; 76%), and/or aprepitant (91/134; 68%). In addition, 22 respondents (16%) indicated using the 5-HT3 receptor antagonist ondansetron to manage treatment-induced nausea in patients.

#### 3.5.3 Diarrhea

Survey respondents were asked in what order they employ four suggested options for managing treatment-related diarrhea (not related to immunotherapy) including: dose reduction, dose delay, change or stop treatment, and using supportive medications to mitigate symptoms. Supportive medications were chosen by 116/133 (87%) as the first choice for managing treatment-related diarrhea, while a nearly identical number of respondents (115/133; 86%) identified changing or stopping treatment as the last option of the 4 to consider.

#### 3.5.4 Peripheral neuropathy

Survey respondents reported the strategies they use to manage treatment-associated peripheral neuropathy in their patients (Supplementary information, Fig. S2). The most frequently chosen options were “dose delays and/or reductions” (124/134; 93%) and “daily medications such as duloxetine, gabapentin” (123/134; 92%). In addition, 36/128 (28%) discuss cryotherapy for peripheral neuropathy with their patients in the adjuvant setting, 22/128 (17%) discuss it during the metastatic setting, and 70/128 (55%) discuss cryotherapy in both treatment settings.

#### 3.5.5 PI3K/AKT/PTEN inhibitors

##### 3.5.5.1 Dermatologic adverse events

A majority of respondents used prophylactic antihistamines when prescribing alpelisib (93/134; 69%), but 41/134 (31%) did not typically follow this recommended practice. Over 90% of respondents used topical corticosteroids (124/134) and/or antihistamines (128/134) to manage treatment associated rash (Supplementary information, Fig. S3).

##### 3.5.5.2 Hyperglycemia

Before starting a patient on alpelisib, 126/134 (94%) respondents would test HbA1c levels, 86/134 (64%) would test fasting glucose values, and 64/134 (48%) would check random glucose values. However, less than half (63/133; 47%) used a specific HbA1c value threshold to determine whether or not to prescribe alpelisib, and 105/134 (78%) would start a patient with controlled type 2 diabetes mellitus on alpelisib. During treatment with alpelisib, 125/133 (94%) would monitor blood glucose levels, and 99/134 (74%) would start monitoring after 1 week of treatment, while a plurality of respondents (59/134; 44%) would monitor blood glucose weekly in these patients. Respondents most frequently use metformin (127/134; 95%) to manage treatment-associated hyperglycemia in patients (Supplementary information, Fig. S4).

#### 3.5.6 Interstitial lung disease

When administering an agent such as T-DXd, 67 of 134 respondents (50%) monitor patients for interstitial lung disease (ILD) with high-resolution computed tomography (CT) every 12 weeks, and 60 (45%) monitor more frequently than that. In diagnosing ILD, most respondents would begin with “clinical grounds” (70/134; 52%), followed by radiographic testing and imaging (66; 49%), and lastly turning to transbronchial biopsy (124; 93%).

## 4 Discussion

The RETRACT survey was developed to explore real-world practice patterns among clinical oncologists who treat patients with HR+/HER2-mBC. The results show that the practice patterns in this sample of oncologists from academic and community treatment centers are largely in alignment with published guidelines.

Respondents favored CDK4/6i plus ET as the first-line treatment, consistent with several widely used treatment guidelines, including for patients who experience disease progression on adjuvant endocrine therapy or with early disease relapse within 12 months of completing adjuvant endocrine therapy [3,4]. Ribociclib, the preferred CDK4/6i, is supported by evidence from several trials showing it is associated with increased OS when added to AI or ET therapy as a first-line treatment [[5], [6], [7],^10^,^15^,^38^].

Most respondents exhaust all ET options before beginning chemotherapy or ADCs, including retreating patients with a CDK4/6i if they progressed on prior CDK4/6i therapy. It should be noted that the supportive trials, MAINTAIN and postMONARCH, evaluated patients switching from palbociclib to ribociclib or abemaciclib [39,40], and there is currently no evidence regarding a switch from either of the agents to the other. The choice of therapy, therefore, should be individualized to each patient and their specific circumstance.

Untried forms of ET may still be effective in patients with endocrine resistance [41], and current guidelines recommend CDK4/6i plus fulvestrant in patients whose disease has progressed during or within12 months after adjuvant ET [3,4,37]. Evidence includes the phase 3 MONARCH 2 trial, which showed benefits of this regimen to PFS and OS in patients who progressed on prior ET [13,14]. Other trials have produced negative results regarding retreating with CDK4/6i inhibitors in patients with ET resistance, although these enrolled fewer patients whose disease progressed during or ≤12 months after adjuvant treatment with CDK4/6i and ET including the MONARCH 3, MONALEESA-3, and PALOMA-3 trials [12,16,42]. Additional factors that could impact the decision of whether to administer an alternative CDK4/6i after progression on ET include Rb mutational status, disease burden, and the sites of disease.

When determining an appropriate second-line treatment, respondents prioritized molecular biomarker status and DOR to first-line therapy, which is appropriate and consistent with current clinical guidelines [3,4]. Respondents’ preference for alpelisib or capivasertib plus fulvestrant for patients with mutated *PIK3CA* is aligned with clinical guidelines [3,4] and results from the phase 3 SOLAR-1 and CAPItello-291 trials, which showed improved median PFS in patients with AKT pathway–altered disease who received capivasertib plus fulvestrant compared to the control group [20].

Responses regarding second-line treatment in patients with mutated *ESR1* were consistent with clinical guidelines [3,4], the FDA-approved indication [42], and evidence from the phase 3 EMERALD trial (NCT03778931) showing elacestrant increased PFS compared with the standard of care in patients with *ESR1* mutations treated with previous ET [22]. The lower level of preference for elacestrant in this setting may reflect lower confidence the efficacy of this SERD in cases with less robust responses to ET in the first line, and some respondents’ answers could have been affected by the timing of this survey relative to when the drug received FDA approval. There is little published data regarding second-line treatment in patients who have mutations in both *PIK3CA* and *ESR1*, but available evidence in patients treated with alpelisib plus fulvestrant shows patients with co-existing *PIK3CA* and *ESR1* mutations had similar responses to this regimen as patients with mutated *PIK3CA* and wild-type *ESR1* [43], suggesting this is the appropriate regimen.

Most patients selected everolimus plus exemestane for second-line treatment in patients with no actionable molecular biomarkers. Data from the phase 3 BOLERO-2 trial (NCT00863655) showed that everolimus plus exemestane resulted in a significantly longer median PFS in patients with HR + advanced BC who had PD on previous NSAI therapy compared to placebo plus exemestane [23,25]. The ESMO and NCCN guidelines recommend this regimen for cases without actionable molecular biomarkers [3,4]. The phase 2 TAMRAD trial suggested PFS and OS were increased by adding everolimus to tamoxifen [44], and the PrECOG trial in patients AI resistant mBC found adding everolimus to fulvestrant significantly improved PFS [26]. However, BOLERO-2 found no significant effect on OS from adding everolimus to exemestane therapy [24].

Regarding visceral crisis, many respondents reported using a definition that differed from the definition used in the ABC5 guidelines [37]. Education around the definition of visceral crisis is needed based on the proportion of patients choosing alternative definitions. However, this definition leaves room for subjectivity and individual judgement in determining visceral crisis, and associated clinical practice will continue to vary between individual providers until more precise criteria are established.

In addition, a substantial minority of respondents indicated they would not use ET plus CDK4/6i therapy in patients with visceral crisis even though this treatment is appropriate, demonstrating a need for education about treating these patients. Evidence includes the phase 2 RIGHT choice trial (NCT03839823), which showed ribociclib plus ET extended PFS and was more tolerable than chemotherapy in patients with investigator-assessed visceral crisis [45]. However, the study excluded some patients with liver metastases, so it is unclear how generalizable the results are to patients with liver-involved visceral crisis. The phase 2 ABIGAIL trial (NCT04603183), likewise, showed improved ORR in patients with aggressive disease, including visceral metastases, who received abemaciclib plus ET compared with paclitaxel [46]. Finally, a retrospective study in the United Kingdom showed patients with visceral crisis or impending visceral crisis treated with CDK4/6i had a significantly longer OS[47], further supporting the use of CDK4/6i in such patients.

Over one-quarter of respondents reported using NGS testing in less than 80% of patients with mBC, suggesting there is an opportunity for education regarding the type and timing of testing. ESMO guidelines recommend NGS testing of advanced BC after resistance to ET, and NGS testing can take the place of germline *BRCA1/2* testing as well [48]. Up-front testing will likely become more relevant since the FDA granted breakthrough status for inavolisib with palbociclib and fulvestrant for *PIK3CA*-mutated, HR+/HER2-, locally advanced or mBC, following recurrence within 12 months of completing adjuvant ET [49]. Inavolisib with palbociclib plus fulvestrant regimen extended median PFS relative to control treatment with no new safety concerns, although all participants had HbA1c levels below 6% [50]. Several biomarkers not included in the survey are also important to test for in mBC, including *RET* fusions [51], *NTRK* fusions in the case of secretory carcinomas [52], as well as high tumor mutational burden (TMB) and high microsatellite instability (MSI) if the use of pembrolizumab is being considered [53,54].

Respondents were aligned with guidelines and/or published evidence regarding strategies for AEs related to ET like switching the ET used, over-the-counter medications, duloxetine, and acupuncture [33]. Most also chose olanzapine, corticosteroids, antiemetics, and the 5-HT3 receptor antagonist ondansetron for treating treatment-associated nausea, which is in alignment with published guidelines and clinical evidence [34,55,56]. Respondents prioritize supportive medications in the management of treatment-related diarrhea before turning to dose reductions or delays, as recommended by ESMO Clinical Practice Guidelines and published clinical experience [57,58]. Clinical guidelines support various interventions for peripheral neuropathy, including duloxetine and cryotherapy [3,35].

Nearly one-third of respondents reported not using prophylactic antihistamines in their patients, but therapies targeting the PI3K/AKT/PTEN signaling pathway, including alpelisib and capivasertib, were been associated with increased rates of grade 3 rash in the SOLAR-1 and CAPItello-291 trials [17,20]. This indicates a need for education on the importance of using of prophylactic anti-rash medications like antihistamines and corticosteroids to prevent unnecessary treatment-associated dermatologic toxicities in patients [58].

The phase 3 DESTINY-Breast04 trial showed T-DXd is associated with an increased rate of ILD or pneumonitis in patients receiving T-DXd (45/371; 12%) relative to those receiving their physician's choice of chemotherapy (1/172; 0.6%) [29], as did the DESTINY-Breast06 trial in patients with HER2-low or HER2-ultralow expressing disease with ILD or pneumonitis in 49/434 (11.3%) of patients receiving T-DXd [28]. Clinical guidelines for monitoring, diagnosing, and managing ILD in patients with breast cancer recommend regular monitoring during treatment with T-DXd including CT scans or high-resolution CT [36,59,60].

### 4.1 Limitations

Clinical oncologists completed this survey on a voluntary basis and may have introduced selection bias among those who chose to participate. The predominance of academic respondents may have affected the results, since it has been shown that practice patterns and perceptions differ among oncologists in these groups [61,62]. However, the anonymous nature of the survey and lack of more granular detail limit the value of subanalyses based on work setting. The sample size used may limit the generalizability of the results. The survey was limited by the rapid progress of developments in this area and did not include the most recently approved treatment options including inavolisib and capivasertib.

## 5 Conclusions

The survey highlighted several areas of educational need including the definition and treatment of visceral crisis, the recommended treatment for patients with endocrine resistance, the routine use of NGS, and the use of prophylactic measures for treatment-related AEs in patients receiving alpelisib. Such unmet educational needs can be addressed through improved education for clinicians as well as through the widespread implementation of multidisciplinary care teams such as tumor boards or specialist breast units, which consist of teams of specialists pooling their expertise to deliver optimal treatment. Reassuringly, the results of the RETRACT survey show that current practice patterns of participating US clinical oncologists are predominantly in alignment with current practice guidelines and available clinical evidence for treating patients with HR+/HER2-mBC.

## CRediT authorship contribution statement

**Hope S. Rugo:** Writing – review & editing, Writing – original draft, Visualization, Supervision, Formal analysis, Conceptualization. **Aditya Bardia:** Writing – review & editing, Writing – original draft, Visualization, Formal analysis, Conceptualization. **William J. Gradishar:** Writing – review & editing, Writing – original draft, Visualization, Formal analysis, Conceptualization. **Erika P. Hamilton:** Writing – review & editing, Writing – original draft, Visualization, Formal analysis, Conceptualization. **Sara A. Hurvitz:** Writing – review & editing, Writing – original draft, Visualization, Formal analysis, Conceptualization. **Komal Jhaveri:** Writing – review & editing, Writing – original draft, Visualization, Formal analysis, Conceptualization. **Reshma Mahtani:** Writing – review & editing, Writing – original draft, Visualization, Formal analysis, Conceptualization. **Sara M. Tolaney:** Writing – review & editing, Writing – original draft, Visualization, Formal analysis, Conceptualization.

## Funding statement

10.13039/100021316AstraZeneca and 10.13039/501100014337Menarini Group provided funding for this project but had no role in manuscript development, review, or approval, nor in the decisions regarding the submission of this manuscript for publication.

## Declaration of competing interest

HSR has received institutional research support from AstraZeneca; Daiichi-Sankyo, Inc.; F. Hoffmann-La Roche AG/Genentech, Inc.; Gilead Sciences, Inc.; Lilly; Merck & Co., Inc.; Novartis Pharmaceuticals Corporation; Pfizer; Stemline Therapeutics, OBI Pharma; and Ambryx, and she has received payments for consultancy and advisory services to Chugai, Puma, Sanofi, Napo, and Mylan. Given her role as Editor, HSR had no involvement in the peer-review of this article and has no involvement in the peer review of this article and has no access to information regarding its peer review. AB has received institutional research support from Pfizer, Novartis, Genentech, Merck, Menarini, Gilead, Sanofi, AstraZeneca/Daiichi-Sankyo, and Eli Lilly, and he has received payments as a consultant to Pfizer, Novartis, Genentech, Merck, Menarini, Gilead, Sanofi, AstraZeneca/Daiichi Sankyo, and Eli Lilly. EPH has received institutional research funding from Abbvie, Acerta Pharma, Accutar Biotechnology, ADC Therapeutics, AKESOBIO Australia, Amgen, Aravive, ArQule, Artios, Arvinas, AstraZeneca, AtlasMedx, BeiGene, Black Diamond, Bliss BioPharmaceuticals, Boehringer Ingelheim, Bristol-Myers Squibb, Cascadian Therapeutics, Clovis, Compugen, Context Therapeutics, Cullinan, Curis, CytomX, Daiichi Sankyo, Dana Farber Cancer Inst, Dantari, Deciphera, Duality Biologics, eFFECTOR Therapeutics, Eisai, Ellipses Pharma, Elucida Oncology, EMD Serono, Fochon Pharmaceuticals, FujiFilm, G1 Therapeutics, Gilead Sciences, H3 Biomedicine, Harpoon, Hutchinson MediPharma, Immunogen, Immunomedics, Incyte, Infinity Pharmaceuticals, Inspirna, InventisBio, Jacobio, Karyopharm, K-Group Beta, Kind Pharmaceuticals, Leap Therapeutics, Lilly, Loxo Oncology, Lycera, Mabspace Biosciences, Macrogenics, MedImmune, Mersana, Merus, Millennium, Molecular Templates, Myriad Genetic Laboratories, Novartis, Nucana, Olema, OncoMed, Oncothyreon, ORIC Pharmaceuticals, Orinove, Orum Therapeutics, Pfizer, PharmaMar, Pieris Pharmaceuticals, Pionyr Immunotherapeutics, Plexxikon, Prelude Therapeutics, Profound Bio, Radius Health, Regeneron, Relay Therapeutics, Repertoire Immune Medicine, Rgenix, Roche/Genentech, SeaGen, Sermonix Pharmaceuticals, Shattuck Labs, Silverback Therapeutics, StemCentRx, Stemline Therapeutics, Sutro, Syndax, Syros, Taiho, TapImmune, Tesaro, Tolmar, Torque Therapeutics, Treadwell Therapeutics, Verastem, Zenith Epigenetics, and Zymeworks. EPH has also received institutional payments for consulting and advisory roles to Accutar Biotechnology, Arvinas, AstraZeneca, Circle Pharma, Daiichi Sankyo, Entos, Gilead Sciences, IQVIA, Janssen, Jazz Pharmaceuticals, Jefferies LLC, Johnson and Johnson, Lilly, Medical Pharma Services, Mersana Therapeutics, Olema Pharmaceuticals, Pfizer, Roche/Genentech, Shorla Pharma, Stemline Therapeutics, Tempus Labs, Theratechnologies, Tubulis, and Zentalis Pharmaceuticals. SAH has received clinical trial grants paid to her institution from Ambrx, Amgen, Arvinas, Astra Zeneca, Bayer, Celcuity, Cytomx, Daiichi-Sankyo, Dantari, Dignitana, Genentech/Roche, G1-Therapeutics, Gilead, Greenwich Life Sciences Inc, GSK, Immunomedics, Eli Lilly, LOXO, Macrogenics, Novartis, OBI Pharma, Orinove, Orum, Pfizer, Phoenix Molecular Designs, Ltd., Pieris, PUMA, Radius, Samumed, Sanofi, Seattle Genetics/Seagen, and Zymeworks. SAH has also received funding for advisory and consulting roles to Briacell, Beigene, Jazz, Boehringer Ingelheim, Menarini, Mersana, Roche, and Arvinas, and she has participated in a data safety monitoring board for InClin and Atossa Therapeutics. SMT has received research funding from Genentech/Roche, Merck, Exelixis, Pfizer, Lilly, Novartis, Bristol Myers Squibb, AstraZeneca, NanoString Technologies, Gilead, SeaGen, OncoPep, Daiichi Sankyo, and Menarini/Stemline, and she has received travel funding from Eli Lilly, Sanofi, Gilead, Jazz, Pfizer, and Arvinas. SMT has also received payments for consulting and advisory roles with Novartis, Pfizer/SeaGen, Merck, Eli Lilly, AstraZeneca, Genentech/Roche, Eisai, Sanofi, Bristol Myers Squibb/Systimmune, Daiichi Sankyo, Gilead, Zymeworks, Zentalis, Blueprint Medicines, Reveal Genomics, Sumitovant Biopharma, Artios Pharma, Menarini/Stemline, Aadi Bio, Bayer, Incyte Corp, Jazz Pharmaceuticals, Natera, Tango Therapeutics, eFFECTOR, Hengrui USA, Cullinan Oncology, Circle Pharma, Arvinas, BioNTech, Launch Therapeutics, Zuellig Pharma, Johnson&Johnson/Ambrx, and Bicycle Therapeutics.

WJG, KJ, and RM have no conflicts to report.
