# Supplementary material for: Expert consensus on treating HR+/HER2- metastatic breast cancer based on real-world practice patterns observed in the RETRACT survey of US oncologists
Source: Breast. 2025 May 3;82:104485. doi: 10.1016/j.breast.2025.104485 (PMC12138406; doi:10.1016/j.breast.2025.104485)
Supplement: Multimedia component 1 [file mmc1.docx]

# Supplemental Information:

**Figure S1: Survey Questions**

Note: In this survey, we use the term “molecular biomarkers” to refer to genes harboring mutations that are potentially clinically actionable (eg, *PIK3CA*, *ESR1*, *BRCA1*). These do not include other biomarkers assessed via immunohistochemistry (eg, ER, PR, HER2).

**General Clinical Practice**

1. In your practice, how many patients with breast cancer do you treat each month?
2. 0-25 patients
3. 26-50 patients
4. 51-100 patients
5. 101-150 patients
6. 151-200 patients
7. >200 patients
8. What percentage of these patients have metastatic disease?
9. 0%-20%
10. 21%-40%
11. 41%-60%
12. 61%-80%
13. 81%-100%
14. What are the key challenges that hinder optimal management of patients with mBC? (select all that apply)
15. Low health literacy among patients
16. Poor treatment adherence
17. Poor adherence to follow-up visits
18. Treatment toxicity
19. Financial barriers (eg, inability to afford treatment)
20. Delays in treatment decision-making
21. Other (please describe): ________________________
22. Which of the following best aligns with your definition of visceral crisis?
23. Severe organ dysfunction, as assessed by signs and symptoms, laboratory studies, and rapid progression of disease
24. Presence of metastases that compromise the function of vital organs
25. Liver metastases with liver dysfunction, lymphangitic pulmonary disease with dyspnea, or significant cytopenias due to bone marrow infiltration
26. Visceral metastases
27. Visceral metastases with multiple sites of disease
28. Short disease-free interval
29. Visceral metastases in a younger aged patient
30. Other (please describe): ________________________
31. Do you agree with the ABC5 definition of primary endocrine resistance: *relapse while on the first 2 years of adjuvant ET, or PD within the first 6 months of first-line ET for ABC, while on ET*?
32. Yes
33. No
    - If not, how would you alter this definition? _____________________

**Biomarker Testing**

1. Please indicate which biomarkers you test for in patients with mBC and the times at which you test for these biomarkers. (select all that apply)

| **Biomarker** | **Tested at diagnosis** | **Tested at progression on 1L therapy** | **Tested at progression after each line of therapy** | **Other (please describe)** |
| --- | --- | --- | --- | --- |
| ER and PR |  |  |  |  |
| HER2 |  |  |  |  |
| Germline mutations, including *BRCA1/2* and *PALB2* |  |  |  |  |
| PD-L1 |  |  |  |  |
| NGS on tissue |  |  |  |  |
| NGS by ctDNA |  |  |  |  |

1. Do you ever test for any of the following biomarkers using single-gene tests, small panels (instead of comprehensive NGS panels), or immunohistochemistry: *PIK3CA*, *AKT1*, *PTEN*, *ESR1*, *BRCA1/2*, *PALB2*, TMB, other?

A. No

B. Yes

Please specify which genes you test for: ____________

| **Sample** | **When you use this type of sample (select all that apply)** | **Why you use this type of sample (select all that apply)** |
| --- | --- | --- |
| Tissue | - At initial diagnosis - After progression - Other | - Gold standard - More accurate representation of current mutational burden - Other (please describe): __________________________ |
| Blood | - At initial diagnosis - After progression - Other | - Easier than obtaining biopsy - Obtained when a biopsy cannot be performed - More accurate representation of current mutational burden - Other (please describe): __________________________ |

1. What proportion of patients with mBC in your practice setting undergo NGS testing?
2. 0%-20%
3. 21%-40%
4. 41%-60%
5. 61%-80%
6. 81%-100%
7. Please indicate when and why you use the following types of samples for molecular biomarker testing in patients with mBC.
8. What are the key barriers you face in molecular biomarker testing for patients with mBC? (select all that apply)
9. Insufficient tissue for analysis
10. Slow turnaround time for the pathology laboratory to find the patient sample block and perform testing or send out for testing
11. Slow turnaround time for receipt of test results
12. Inadequate reimbursement/lack of reimbursement for testing
13. Lack of clear guidance regarding testing
14. Difficulty in interpreting test results and translating them into patient care
15. Ambiguity on when to test
16. “Waiting anxiety” among patients
17. Lack of targetable markers
18. Other (please describe)

**1L Treatment Decisions**

1. What percentage and types of patients in your practice with HR+/HER2- mBC receive the following as 1L treatment?

| **Treatment** | **Percentage that receive in 1L** | **Type of patient/patient profile (please describe)** |
| --- | --- | --- |
| CDK4/6i + AI | 1. 0%-20% 2. 21%-40% 3. 41%-60% 4. 61%-80% 5. 81%-100% | [open field] |
| CDK4/6i + fulvestrant | 1. 0%-20% 2. 21%-40% 3. 41%-60% 4. 61%-80% 5. 81%-100% | [open field] |
| ET monotherapy | 1. 0%-20% 2. 21%-40% 3. 41%-60% 4. 61%-80% 5. 81%-100% | [open field] |
| Conventional chemotherapy | 1. 0%-20% 2. 21%-40% 3. 41%-60% 4. 61%-80% 5. 81%-100% | [open field] |
| Other (eg, radiation, ADCs, etc) | 1. 0%-20% 2. 21%-40% 3. 41%-60% 4. 61%-80% 5. 81%-100% | [open field] |

1. Which CDK4/6i do you prefer to use in the 1L setting in mBC?
2. Abemaciclib
3. Palbociclib
4. Ribociclib
5. In what setting and for what types of patients do you choose **not** to treat with CDK4/6i + ET in the 1L setting? (select all that apply)
6. Patients in visceral crisis (after chemotherapy induction as needed)
7. Patients with visceral metastasis
8. Patients with CNS metastasis
9. Patients with bone-only metastasis
10. Patients with de novo metastatic disease
11. Patients with <6-12 months DFI following adjuvant CDK4/6i-containing therapy
12. Patients with >5 years DFI following adjuvant therapy
13. Other (please describe): ___________
14. Approximately what proportion of your patients with HR+/HER2- mBC present with visceral crisis?
15. 0%-5%
16. 6%-10%
17. 11%-15%
18. 16%-20%
19. >20%
20. What is your preferred treatment regimen for patients with HR+/HER2- visceral crisis (with clinically significant organ dysfunction)?
21. CDK4/6i + ET
22. Chemotherapy
23. Chemotherapy followed by CDK4/6i + ET as maintenance
24. Other (please describe): ______________
25. If a patient with HR+/HER2- mBC received a CDK4/6i in the adjuvant setting and progressed <6 months after completing treatment (while still on adjuvant ET), would you prescribe CDK4/6i + a different ET in the 1L mBC setting?
26. Yes
27. No
28. Not sure

16a. If you answered yes, would you use the same or a different CDK4/6i?

- 1. Same

B. Different

1. If a patient with HR+/HER2- mBC received a CDK4/6i in the adjuvant setting and progressed <12 months after completing treatment (while still on adjuvant ET), would you prescribe CDK4/6i + a different ET in the 1L mBC setting?
2. Yes
3. No
4. Not sure

17a. If you answered yes, would you use the same or a different CDK4/6i?

- 1. Same

B. Different

1. If a patient received a CDK4/6i in the adjuvant setting and progressed >12 months after completing treatment with the CDK4/6i, would you prescribe CDK4/6i + ET in the 1L mBC setting?
2. Yes
3. No
4. Not sure

18a. If you answered yes, would you use the same or a different CDK4/6i?

- 1. Same

B. Different

**2L+ Treatment Decisions**

19. What proportion of your patients with HR+/HER2- mBC receive some form of 2L treatment (either with ET or chemotherapy)?

1. 0%-20%
2. 21%-40%
3. 41%-60%
4. 61%-80%
5. 81%-100%
6. One a scale of 1 to 5 (1 = extremely important; 5 = not important), please rate the importance of the following factors for determining the best 2L treatment option for patients following progression on 1L CDK4/6i + ET.
7. Duration of response to 1L therapy
8. Molecular biomarker status (eg, *PIK3CA* mutation, *ESR1* mutation)
9. Other biomarker status (ER/PR expression, HER2 expression)
10. Site of progression/metastasis (eg, bone only, visceral)
11. Clinical/pathological features (eg, burden of disease, pace of progression)
12. Availability of a clinical trial
13. Other; please describe: __________________

1. Do you think it is clinically meaningful to differentiate patients as having “endocrine refractory” or “endocrine sensitive” disease to guide clinical decision-making?
2. No
3. Yes
   - If you answered yes, what guidance/criteria do you follow?
     - ABC5 or ESMO definitions (primary resistance, secondary resistance) (eg, see <https://www.annalsofoncology.org/article/S0923-7534(20)42460-3/fulltext>)
     - Clinical judgement based on the patient profile (please describe the key considerations): ____________________
     - Other guidelines (please describe): ______________
   - If you answered yes, how does this impact your treatment recommendations for a patient with refractory disease?
     - I would use chemotherapy rather than sequential ET.
     - I would be more likely to enroll the patient in a clinical trial of ET + targeted therapy.
     - I would only use ET + targeted therapy and not ET alone.
     - I would not use ET maintenance therapy.
4. Do you usually try to exhaust all possible ET-based treatment options (with or without targeted agents) before transitioning patients to chemotherapy/ADCs?
5. Yes
6. No
7. Which treatments do you prefer to use in the 2L setting in the following patient tumor types, assuming the patient received 1L CDK4/6i + ET? (select all that apply)

| **Patient Profile** | **Treatment (if you select “clinical trial” as an option, please select at least 1 other option that you would consider if a clinical trial was not available)** |
| --- | --- |
| *PIK3CA* mutation | 1. Different CDK4/6i + ET 2. Alpelisib + fulvestrant 3. Everolimus + exemestane or fulvestrant (or other ET) 4. Fulvestrant monotherapy 5. Chemotherapy/ADC 6. Clinical trial 7. Other (please describe) |
| *ESR1* mutation and duration of response >12 months on 1L therapy | 1. Different CDK4/6i + ET 2. Everolimus + exemestane or fulvestrant (or other ET) 3. Elacestrant 4. Chemotherapy/ADC 5. Clinical trial 6. Other (please describe) |
| *ESR1* mutation and duration of response <12 months on 1L therapy | 1. Different CDK4/6i + ET 2. Everolimus + exemestane or fulvestrant (or other ET) 3. Elacestrant 4. Chemotherapy/ADC 5. Clinical trial 6. Other (please describe) |
| Both *PIK3CA* and *ESR1* mutation | 1. Different CDK4/6i + ET 2. Alpelisib + fulvestrant 3. Everolimus + exemestane or fulvestrant (or other ET) 4. Fulvestrant monotherapy 5. Elacestrant 6. Chemotherapy/ADC 7. Clinical trial 8. Other (please describe) |
| No molecular biomarkers | 1. Different CDK4/6i + ET 2. Everolimus + exemestane or fulvestrant (or other ET) 3. Chemotherapy/ADC 4. Clinical trial 5. Other (please describe) |

1. What proportion of your patients with *PIK3CA*-mutated HR+/HER2- mBC receive alpelisib + fulvestrant as second/subsequent-line therapy?
2. 0%-20%
3. 21%-40%
4. 41%-60%
5. 61%-80%
6. 81%-100%
7. What proportion of your patients with *ESR1*-mutated HR+/HER2- mBC receive single agent elacestrant as second/subsequent-line therapy?
8. 0%-20%
9. 21%-40%
10. 41%-60%
11. 61%-80%
12. 81%-100%
13. What proportion of your patients with HR+/HER2- mBC and no targetable molecular biomarkers receive everolimus + exemestane or fulvestrant (or another ET) as second/subsequent-line therapy?
14. 0%-20%
15. 21%-40%
16. 41%-60%
17. 61%-80%
18. 81%-100%

**Subsequent-line Therapy**

1. What proportion of your patients with HR+/HER2- mBC receive some form of 3L treatment?
2. 0%-20%
3. 21%-40%
4. 41%-60%
5. 61%-80%
6. 81%-100%
7. What proportion of your patients with HR+/HER2- mBC receive some form of 4L treatment?
8. 0%-20%
9. 21%-40%
10. 41%-60%
11. 61%-80%
12. 81%-100%
13. What proportion of your patients with HR+/HER2- mBC receive some form of 5L+ treatment?
14. 0%-20%
15. 21%-40%
16. 41%-60%
17. 61%-80%
18. 81%-100%
19. Do you ever prescribe an ET-based regimen after a patient has received chemotherapy/ADC?
20. No
21. Yes

- Under what circumstances? Please describe:_______________

1. Yes, only as maintenance after chemotherapy response

Note: Treatment-related or treatment-associated toxicities refer to those associated with immunotherapy, CDK4/6i monotherapy and combinations, conventional chemotherapy, ET monotherapy and combinations, or ADCs.

**Management and Monitoring of Toxicities**

1. What strategies do you employ to manage intolerable adverse events associated with ET? (select all that apply)

A. NSAIDS/OTC medications

B. Duloxetine

C. Switch to a different ET

D. Other; please describe: ______________________

32. In what order do you employ the following strategies to manage treatment-related diarrhea (not related to immunotherapy)?

Please order from 1 to 4

1. Dose reduction
2. Dose delay
3. Change or stop treatment
4. Supportive medications to mitigate symptoms

33. Do you use prophylactic antihistamines in patients receiving alpelisib or other targeted therapy associated with rash?

A. Yes

B. No

34. How do you typically manage treatment-associated rash in your patients? (select all that apply)

A. Use of antihistamines

B. Topical corticosteroids

C. Systemic corticosteroids

D. Dose reduction

E. Dose delay

F. Stop treatment

35. If you are planning to start alpelisib, what laboratory values do you check in advance? (Check all that apply)

A. HbA1c

B. Fasting glucose

C. Random glucose

36. Do you have an HbA1c cutoff for starting alpelisib

A. No

B. Yes

If yes, what is your cutoff? __________________

37. Would you start a patient with controlled type 2 diabetes on alpelisib?

A. No

B. Yes

38. Do you monitor blood glucose levels in patients prescribed alpelisib?

A. No

B. Yes

When do you start monitoring?

After 1 week

After 2 weeks

After 3 weeks

After ≥4 weeks

How frequently do you monitor?

Once a week

Once every 2 weeks

Once every 3 weeks

Once a month

39. How do you typically manage treatment-associated hyperglycemia in your patients? (select all that apply)

A. Reduced carbohydrate diet

B. Metformin

C. SGLT2 inhibitor

D. Pioglitazone

E. Insulin

F. Dose reduction

G. Dose delay

H. Stop treatment

40. How do you manage peripheral neuropathy in your patients? (select all that apply)

1. Dose delays and/or reductions
2. Change the treatment regimen
3. Daily medications such as duloxetine, gabapentin
4. As needed pain medications
5. A combination of therapy and medication
6. Physical therapies (acupuncture, cryotherapy, etc.)

40a. If you routinely discuss cryotherapy with patients, in which setting do you do so:

- 1. Adjuvant
  2. Metastatic
  3. Both adjuvant and metastatic

41. How do you manage treatment-related nausea in your patients? (select all that apply)

A. Olanzapine as a rescue medicine

B. Nonpharmacologic agents such as relaxation and behavioral therapy

C. Change in regimen

D. Corticosteroids

E. Aprepitant as a rescue medicine

F. Combination of above: _______________

42. When administering an agent associated with interstitial lung disease (ILD)/pneumonitis (eg, trastuzumab deruxtecan), how often do you monitor patients with high-resolution CT?

A. Every 6 weeks

B. Every 9 weeks

C. Every 12 weeks

D. Every 16 weeks

E. Other; please describe: __________________________

43. What steps do you take in diagnosing treatment-induced ILD/pneumonitis in your patients? (Please order from 1-3)

A. Transbronchial Biopsy (TBB)

B. Clinical Grounds

C. Radiographic Tests and Imaging

44. For what proportion of your patients do you consult with other specialists (eg, endocrinologists, dermatologists, pulmonologists) for management of treatment-related toxicities?

A. 0-5%

B. 6-10%

C. 11-15%

D. 16-20%

E. >20%

**Supplemental Figure S2: Respondents indicated how they manage peripheral neuropathy in their patients. Multiple answers were permitted (n = 134).**

**Supplemental Figure S3: Respondents indicated how they manage treatment- associated rash in their patients. Multiple answers were permitted (n = 134).**

**Supplemental Figure S4: Respondents indicated how they manage treatment-associated hyperglycemia in their patients. Multiple answers were permitted (n = 134).**
